# Enterovirus 104 Infection in Adult, Japan, 2011

**DOI:** 10.3201/eid1805.111890

**Published:** 2012-05

**Authors:** Atsushi Kaida, Hideyuki Kubo, Jun-ichiro Sekiguchi, Atsushi Hase, Nobuhiro Iritani

**Affiliations:** Osaka City Institute of Public Health and Environmental Sciences, Osaka, Japan

**Keywords:** Enterovirus 104, viruses, acute respiratory tract infection, adult, Japan

**To the Editor:** Human enterovirus (HEV) C (family *Picornaviridae*, genus *Enterovirus*) consists of 3 types of poliovirus (1, 2, and 3), 9 types of coxsackievirus A (CV-A1, 11, 13, 17, 19, 20, 21, 22, and 24), and 9 types of enterovirus (EV) (95, 96, 99, 102, 104, 105, 109, 113, and 116) (www.picornaviridae.com/enterovirus/hev-c/hev-c.htm). EV-104 was first identified in 2009 in Switzerland in 8 children who had pneumonia or acute otitis media (*1*). To our knowledge, there has been only 1 other report of EV-104, detected in Italy in 3 adults and 2 children who had upper respiratory tract infection (RTI) (*2*). We report the detection of a novel EV-104 strain in an adult with upper RTI in Japan.

In February 2011, a nasal swab specimen was collected from a 36-year-old immunocompetent man in Japan who had rhinorrhea, cough, pharyngitis, and fever (38.3°C). The sample underwent viral nucleic acid extraction and cDNA synthesis (*3*) and was PCR screened for HEV and human rhinovirus [HRV] by using primers EVP4 and OL68-1, which detect viral protein (VP) 4/VP2 gene in HEV and HRV as amplicons of ≈650 and 530 bp, respectively (*4*). Unexpectedly, an amplicon of ≈600 bp was generated.

To identify this amplicon, sequencing and BLAST analysis (www.ncbi.nlm.nih.gov) was conducted and yielded a 522-nt sequence with sequence similarity to EV-104 (94.4% identity with the prototype strain CL-12310945; 7,229 nt [GenBank accession no. EU840733]). The sequence similarity corresponded to nt 633–1154 of the novel strain, which was designated AK11 (7,408 nt; GenBank accession no. AB686524).

The complete genome sequence of AK11 was determined as follows: cDNA was synthesized by using sequence-specific primers and amplified as 4 fragments (nt 1–494, 65–3852, 1975–3852, and 3284–7408 with poly A). End-specific nucleotide sequences were determined by using the 5′ RACE system (Rapid Amplification of cDNA Ends; Invitrogen, Life Technologies Corp., Carlsbad, CA, USA) and 3′ RACE by using primer TX30SXN (*5*).

The genome and predicted amino acid sequences of AK11 were compared with those of CL-12310945, the only EV-104 strain for which a large part of the genome sequence is available. This analysis showed that CL-12310945 is shorter than AK11 at both termini. Specifically, nt 1–64 (corresponding to the 5′ untranslated region [UTR]) and nt 7291–7408 (corresponding to part of the 3D gene and the 3′ UTR) of AK11 were not sequenced from CL-12310945.

The identities between the strains were calculated by using BioEdit version 7.09 (www.mbio.ncsu.edu/bioedit/bioedit.html), with results as follows: 5′ UTR (partial sequence 95.0% nt identity; amino acid identity not applicable), VP4 (95.2% nt, 100% aa), VP2 (95.6% nt, 96.3%), VP3 (95.2% nt, 99.6% aa), VP1 (96.2% nt, 99.0% aa), 2A (96.2% nt, 99.3% aa), 2B (94.8% nt, 100% aa), 2C (92.0% nt, 99.1% aa), 3A (83.3% nt, 94.3% aa), 3B (84.8% nt, 90.9% aa), 3C (84.5% nt, 94.5% aa), and 3D (partial sequence 84.7% nt, 93.3% aa). The 3′ UTR was not analyzed.

Phylogenetic analysis of VP1 sequences among HEV-C viruses showed that AK11 clusters with CL-12310945 and is genetically close to CV-A1, CV-A19, CV-A22, and EV-109 (Figure). These results are consistent with reported results (*6*). Virus isolation, attempted by using Vero and RD-18S cells, was unsuccessful. This result is consistent with previous EV-104 reports, wherein the virus could not be grown or isolated (*1**,**2*).

**Figure:**
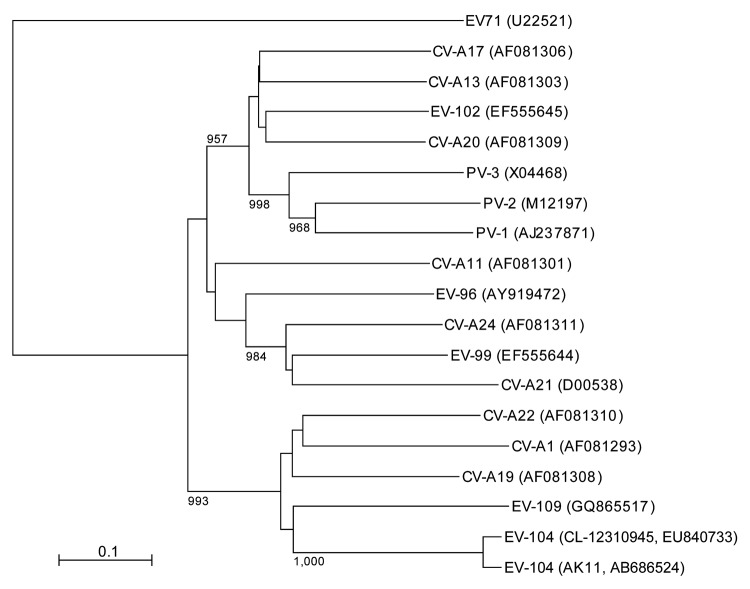
Phylogenetic tree of human enterovirus C and viral protein (VP) 1 sequences constructed by using the neighbor-joining method. Complete VP1 gene sequences in enterovirus (EV) 104 (888 nt, corresponding to nt 2461–3348 of novel strain AK11) and other human enterovirus C viruses were aligned by using ClustalX version 2 (www.clustal.org). Sequences in EV-95, EV-105, EV-113, and EV-116 were not available from the database. Genetic distances between sequences were calculated by using the Kimura 2-parameter method. Bootstrap values >950 from 1,000 replicates are shown at the nodes. GenBank accession numbers for strains used in this analysis are shown in parentheses. Scale bar indicates nucleotide substitutions per site. CV-A, coxsackievirus A; PV, poliovirus. EV-71 was used as outgroup.

To determine the presence of other respiratory viruses in this patient, the EV-104–positive specimen was tested by using real-time PCR for any of 17 other viruses (human metapneumovirus, respiratory syncytial virus, human parainfluenza virus types 1–4, human bocavirus, human coronavirus [229E, OC43, HKU1, NL63], influenza virus [A, pandemic (H1N1) 2009, B, C], human adenovirus, and HRV). No other viruses were detected (data not shown). This result indicates that EV-104 was associated with upper RTI in this patient. During the 2 months in which the EV-104–positive sample was collected, influenza A virus, HRV, and respiratory syncytial virus were most frequently detected in other patients, and no enterovirus was observed in other specimens from persons with RTI.

EV-104 detection is rare (5/1,500 [0.3%] for a 1-year study in Italy [*1*]; 8/1,592 [0.5%] for a 10-year study in Switzerland [*2*]). As part of a virus surveillance program in Osaka City, Japan, during November 2010–October 2011, a total of 645 respiratory tract specimens were collected from children with RTI (360 male, 285 female; age 0–59 months, mean ± SD 18.9 ± 13.8 months) and subjected to PCR by using EVP4 and OL68-1 primers. No EV-104 was detected. In 2 previous studies in Japan, we detected no EV-104 in 764 specimens from patients with RTI during November 2008 and October 2010 (*3**,**7*); therefore, we have found EV-104 in only 1 (0.07%) of 1,410 samples tested.

Infrequent detection and insensitivity to cell culture contribute to the rarity of EV-104 identification. However, given the lack of contact between EV-104–positive patients in Italy and Switzerland, more RTI patients might actually be carrying EV-104 than testing has indicated. The collection of additional EV-104 strains and associated epidemiologic and virologic information will help clarify the role of this virus in RTI.
